# Enzyme replacement therapy and white matter hyperintensity progression in Fabry disease

**DOI:** 10.1212/WNL.0000000000006316

**Published:** 2018-10-09

**Authors:** James D. Stefaniak, Laura M. Parkes, Adrian R. Parry-Jones, Gillian M. Potter, Andy Vail, Ana Jovanovic, Craig J. Smith

**Affiliations:** From the Greater Manchester Comprehensive Stroke Centre, Clinical Sciences Building (J.D.S., A.R.P.-J., C.J.S.), Department of Neuroradiology, Greater Manchester Neurosciences Centre (G.M.P.), and The Mark Holland Metabolic Unit (A.J.), Salford Royal NHS Foundation Trust, Manchester Academic Health Science Centre, Salford; and Neuroscience and Aphasia Research Unit, Division of Neuroscience and Experimental Psychology (J.D.S.), Division of Neuroscience and Experimental Psychology (L.M.P.), Division of Cardiovascular Sciences, School of Medical Sciences (A.R.P.-J., C.J.S.), and Centre for Biostatistics, Division of Population Health, Health Services Research and Primary Care (A.V.), University of Manchester, Manchester Academic Health Science Centre, UK.

## Abstract

**Objective:**

To explore the association between enzyme replacement therapy (ERT), clinical characteristics, and the rate of progression of white matter hyperintensities (WMH) in patients with Fabry disease (FD).

**Methods:**

Patients with a confirmed diagnosis of FD, aged 18 years or older, participating in an existing FD observational study (NCT00196742), with at least 2 serial MRI brain scans at least 2 years apart for the period between December 2006 and August 2016 were included in this cohort study. Total WMH volume was estimated for each image using a semiautomated procedure. We performed linear regression to calculate the primary outcome measure of WMH change rate for each participant. Associations between ERT, clinical characteristics, and the primary outcome were explored using multiple linear regression.

**Results:**

Eight hundred sixty-three MRI time points were analyzed for the 149 included participants. Age (*p* < 0.0005; increasing age associated with faster WMH progression), total cholesterol (*p* = 0.03; increasing total cholesterol associated with slower WMH progression), and a history of peripheral pain (*p* = 0.02; peripheral pain associated with faster WMH progression) were independently associated with WMH change rate in the primary analysis. We did not find an association between “ERT at any point between baseline and final MRI” and WMH change rate (*p* = 0.22).

**Conclusion:**

In a large cohort of patients with FD, we did not find an association between ERT and WMH progression, while higher total cholesterol was associated with slower WMH progression. Further research is needed into the pathogenesis and treatment of cerebrovascular disease in this rare condition.

Fabry disease (FD) is an X-linked lysosomal storage disorder caused by α-galactosidase A deficiency due to mutations in the *GLA* gene.^1^ Individuals with FD are at high risk of premature cerebrovascular disease, including early-onset ischemic stroke.^2^ White matter hyperintensities (WMH) on MRI are thought to represent cerebral microangiopathic changes and have a reported prevalence of 42% to 81% in FD.^3–9^ WMH have been associated with left ventricular hypertrophy,^8^ cardiomyopathy,^10^ previous stroke,^8,10^ and conventional vascular risk factors^6^ in patients with FD and with an increased risk of death,^11^ dementia,^12^ stroke,^13^ and cognitive decline^14,15^ in the general population. A better understanding of factors influencing WMH progression in patients with FD could therefore provide insight into preventing these sequelae.

Enzyme replacement therapy (ERT) was, until the recent approval of migalastat (Galafold; Amicus Therapeutics, Cranbury, NJ) in the European Union,^16^ the only approved disease-modifying therapy for patients with FD. While ERT significantly reduces microvascular endothelial deposits of glycosphingolipids^17^ and beneficial effects on cardiac and renal parameters have been reported,^18^ the influence of ERT on WMH progression or stroke risk is unclear.^17,18^ In particular, it has been suggested that ERT might be unable to influence progression of cerebrovascular disease in FD because of the relative impermeability of the blood-brain barrier to its passage.^19–21^ The aim of this study was therefore to explore the association between ERT, and other clinical factors, on the rate of progression of WMH in patients with FD.

## Methods

### Participants and setting

As part of clinical follow-up at the Salford Royal Foundation Trust (SRFT) Mark Holland Metabolic Unit, patients with FD undergo annual investigations including blood tests and MRI brain scans and are consented into an ongoing international database that monitors the natural history and outcomes of patients with FD.

To be included in this observational study, patients had to have a confirmed diagnosis of FD (based on α-galactosidase A activity and genotype); be 18 years or older; undergo follow-up at SRFT; participate in the existing FD database; and have at least 2 serial MRI brain scans at least 2 years apart available on the SRFT Picture Archive Communication System (PACS) for the period between December 2006 and August 2016. One hundred sixty-seven patients fulfilled these criteria. In addition, included patients had to have data available on the SRFT FD database for the following clinical characteristics: age at baseline MRI; sex; history of peripheral pain; ERT at any time between baseline and final MRI; and vascular risk factors that might confound any relationship between ERT and WMH progression, including ever smoked at any time between baseline and final MRI, diabetic at any time between baseline and final MRI, hypertension at any time between baseline and final MRI, estimated glomerular filtration rate (eGFR) at baseline MRI, total cholesterol at baseline MRI, and ever had a stroke before baseline MRI. Eighteen of 167 patients were excluded because they had missing data for one or more of these clinical characteristics. One hundred forty-nine participants were therefore included who had a complete dataset for each variable of interest in the primary statistical analysis.

### Data collection

The study commenced in August 2016; archived MRIs were available on PACS from December 2006. The SRFT FD database was accessed by a research data clerk independent of the study team to identify eligible patients and extract data for the above clinical characteristics. MRI brain scans from PACS between December 2006 and August 2016 were anonymized and extracted onto an encrypted external hard drive for WMH analyses. The 149 included participants had a total of 863 MRIs during the period between December 2006 and August 2016 (median [interquartile range, IQR] of 6 [4] MRI scans over median [IQR] 6.1 [3.7] years per participant with median [IQR] interval between consecutive MRI scans of 13 [3] months).

### MRI analysis

MRI brain analysis was undertaken by J.D.S. under the supervision of L.M.P. and G.M.P. All MRI scans included a T2-weighted fluid-attenuated inversion recovery (FLAIR) image. WMH were defined according to standard criteria as white matter regions of hyperintense T2-FLAIR signal without cavitation.^22^ Volumetric quantification was performed because of its greater reliability and sensitivity for longitudinal WMH measurements compared to visual ratings scales.^23,24^ The “gold standard” approach to WMH segmentation for volumetric quantification is manual tracing, but this is very time-consuming. Automated methods exist, such as the “lesion segmentation toolbox” in Statistical Parametric Mapping software,^25^ but at the time of analysis, to calculate WMH volumes, this required a structural T1-weighted MRI along with a corresponding T2-weighted FLAIR MRI. Since the imaging protocols used in the SRFT FD database did not capture T1-weighted images, T2-weighted FLAIR images were analyzed using semiautomated volumetric software, “uom_overlay_editor,” which was developed at the University of Manchester^26^ and does not require the presence of a corresponding T1-weighted image. A variant of this software has been used previously.^27^ J.D.S. quantified total WMH volumes for all 863 available MRIs in random order while blinded to clinical characteristics.

**Figure 1:**
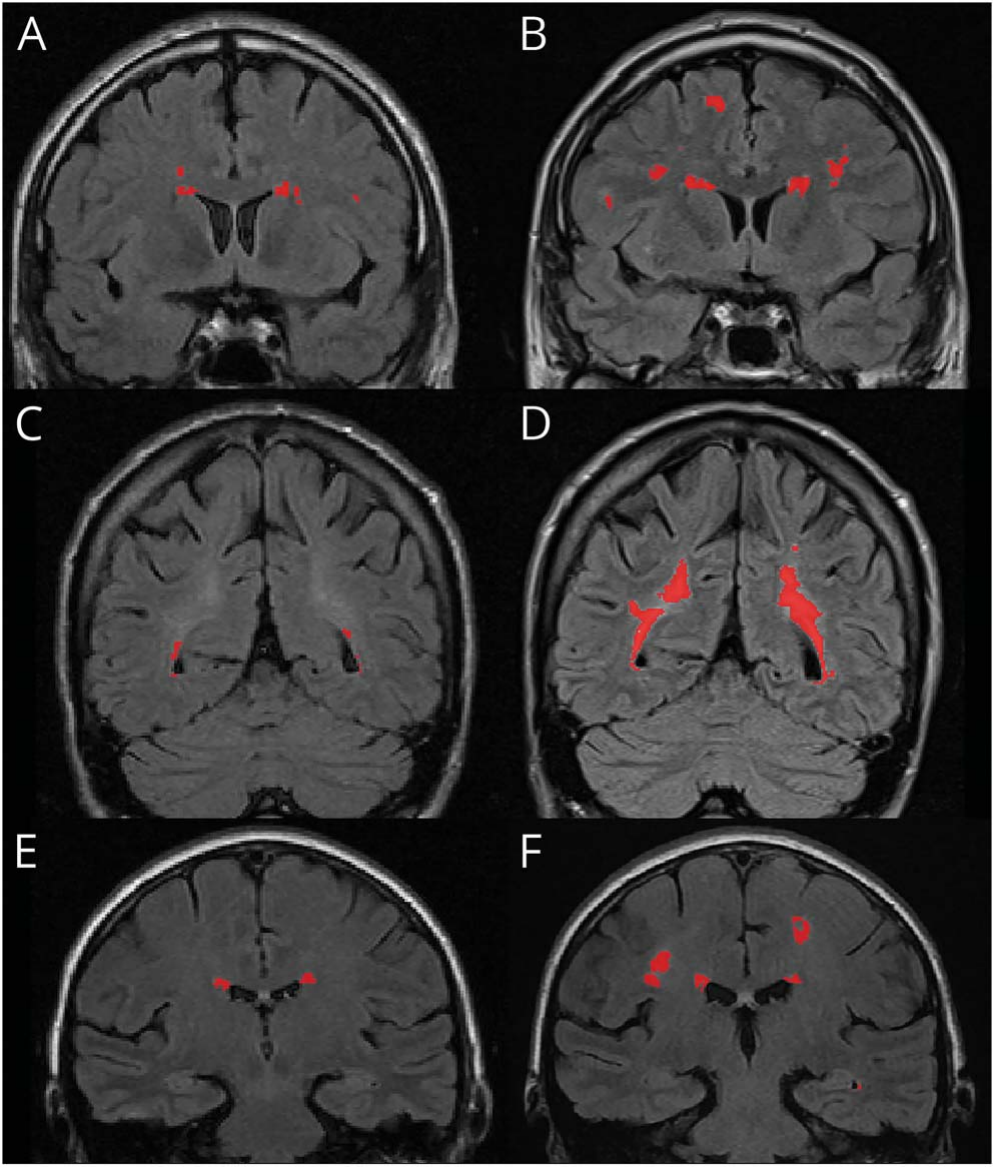
WMH progression in 3 patients with Fabry disease Coronal T2-weighted FLAIR MRIs with WMH highlighted in red by uom_overlay_editor software. MRIs were selected to demonstrate the WMH lesion mask created by uom_overlay_editor in patients with WMH progression. (A and B) FLAIR MRIs from the same participant taken after a 6-year follow-up period. (C and D) FLAIR MRIs from a second participant taken after an 8-year follow-up period. (E and F) FLAIR MRIs from a third participant taken after a 6-year follow-up period. FLAIR = fluid-attenuated inversion recovery; WMH = white matter hyperintensity.

The uom_overlay_editor software included 2 options to create a binary mask over each 2-dimensional slice of the FLAIR image for WMH identification. The first allowed the user to encircle a WMH and then threshold the signal amplitude within that circle until the desired delineation of the WMH was achieved. This method was used for areas of diffuse WMH. The second option allowed the user to select a small region in the center of a WMH, which could then be automatically grown until it reached the edge of the WMH. The system computes the distribution of the intensities in the current region and adds neighboring voxels if they appear to come from the same distribution. This was used for discrete WMH. A binary mask identifying WMH was repeated on each slice for total WMH volume calculation (figure 1).

To assess the accuracy of the uom_overlay_editor software, J.D.S. used MRIcro software^28^ to create lesion segmentation masks manually for WMH and calculate the “gold standard” WMH volume in a randomly selected sample of 20 T2-FLAIR images while blinded to clinical characteristics and the WMH volumes calculated by uom_overlay_editor. Each manually created lesion segmentation mask was converted into a gold standard WMH volume by multiplying the number of voxels in the mask by the voxel volume. The mean (SD) manually calculated total WMH volumes of these 20 T2-FLAIR images was 7.3 (13.8) cm^3^. The intraclass correlation coefficient based on a random-effects model assessing absolute agreement between the manually calculated and uom_overlay_editor calculated total WMH volumes was 0.99, indicating excellent absolute agreement between the uom_overlay_editor WMH volume quantification method and the gold standard of manual WMH tracing. The within-subject SD between the uom_overlay_editor and manually segmented volumes was calculated as per standard methods^29^ at 0.31 cm^3^.

To assess the intrarater measurement error, 20 T2-FLAIR images were randomly selected out of the 863 included in the analysis and their WMH volumes were measured a second time while blinded to clinical characteristics and the original WMH volume. The within-subject SD on the measured WMH volumes was calculated as per standard methods^29^ at 0.17 cm^3^. The upper 95% confidence interval of the intrarater measurement error therefore equals 1.96 × (within-subject SD) = 0.34 cm^3^. The 95% confidence interval on the cube-root volume estimate, using the same methods, is 0.073 cm.

### Primary statistical analysis

We used SPSS version 24 (IBM Corp., Armonk, NY) and defined statistical significance as *p* < 0.05 with Bonferroni correction applied to the significance thresholds. Total WMH volumes (cm^3^) were positively skewed and were therefore cube-root transformed.

For each participant, we performed linear regression with the dependent variable being the cube root of total WMH volume (cm^3^) at each MRI time point and the independent variable being the time (years) from baseline MRI to each MRI time point. This yielded the primary outcome measure of WMH change rate for each participant. Linear regression incorporating data from all MRIs performed on a particular participant during the studied period (December 2006 to August 2016) was used because this incorporated data from the median 6 MRIs performed on each of the 149 participants, therefore increasing the accuracy of our results over a simple rate of WMH change analysis based on the difference in total WMH volume between 2 MRI time points.

Unifactorial comparisons between the participant characteristics outlined above and the WMH change rate were initially performed to explore candidate factors. We then performed multiple linear regression with WMH change rate as the dependent variable and all the above participant characteristics as independent variables. In fitting this multifactorial model, we undertook regression diagnostics for collinearity.

### Post hoc statistical analyses

Following the results of our primary statistical analysis, we performed additional post hoc analyses. To check whether incorporation of information regarding duration of ERT treatment would alter the significance of our results, we performed post hoc analysis 1, in which the duration of ERT treatment by the time of the final MRI (in months) was calculated for all 149 participants included in the primary statistical analysis. All participants who did not have ERT at any time between the baseline and final MRI had also not been on ERT before the baseline MRI; these participants were categorized as having a treatment duration of 0 months by the time of the final MRI. Multiple linear regression against WMH change rate was repeated with the same independent variables as in the primary statistical analysis, except “ERT at any time between baseline and final MRI” was replaced with “duration of ERT at final MRI.”

In response to the unexpected finding that increasing total cholesterol was associated with slower WMH progression, we performed post hoc analysis 2 in order to better elucidate the relationship between cholesterol, statin use, and WMH progression in our cohort. One hundred seventeen of the 149 included participants had data available regarding statin use; using this subgroup, we performed multiple linear regression with WMH change rate as the dependent variable. Since all participants who had a previous stroke were also on a statin, there would be collinearity between statin use and stroke history if both were independent variables in the same multiple regression analysis. We therefore created 2 additional independent variables for inclusion in post hoc analysis 2: (1) “statin–no stroke” (Was the participant on a statin at any time between baseline and final MRI but without having a stroke before baseline MRI?), and (2) “statin-stroke” (Was the participant on a statin as well as having a stroke before baseline MRI?).

To check whether ERT influences the rate of WMH progression in young participants, as has been suggested in previous studies,^8,10^ we performed post hoc analysis 3. Eighty-one of the 149 included participants were younger than 40 years at baseline; using this subgroup, we performed multiple linear regression with WMH change rate as the dependent variable and the above participant characteristics as independent variables, not including statin use or diabetes history (because no patients younger than 40 years had diabetes).

Because of the absence of a high-resolution T1 image covering the full intracranial volume for each person, we did not calculate intracranial volume and therefore were not able to normalize WMH volumes to account for differences in head sizes. It is possible that differing head size could influence the WMH change rate. However, we would expect this to be reflected in the baseline WMH volume (i.e., larger head size, larger baseline WMH volume). To check whether greater baseline WMH volume was associated with faster WMH progression, we performed post hoc analysis 4. We performed multiple linear regression on the 149 participants included in the primary statistical analysis; the dependent variable was WMH change rate, and we included all measured participant characteristics from the primary statistical analysis, as well as the cube root of the baseline MRI total WMH volume (in cm^3^) (“baseline total WMH volume”), as independent variables.

As the MRI scans used were acquired for clinical purposes, different scanners and different image acquisition parameters were used throughout the duration of this study. The range of MRI scanners and T2-FLAIR image acquisition parameters used are in the data available from Dryad (tables e-1 and e-2, doi.org/10.5061/dryad.9271vq7). The 3 scanning parameters hypothesized to be the most important potential sources of variation in total WMH volume calculated from T2-FLAIR MRI scans were the scan orientation, voxel volume, and interslice gap. To investigate whether these 3 variables caused systematic differences in the total WMH volume calculated from corresponding T2-FLAIR MRI scans, we performed post hoc analysis 5. Multiple linear regression was performed with the cube root of the total WMH volume (cm^3^) of the baseline MRI scan as the dependent variable and scan orientation, voxel volume, and interslice gap as independent variables. Because of nonindependence of WMH volumes between MRIs taken at different time points on the same participant, only the cube root of the total WMH volume of the baseline MRI scan was used for each of the 149 included participants.

### Data availability

Anonymized data not published within this article will be shared by request from any qualified investigator.

### Standard protocol approvals, registrations, and patient consents

Patients with FD at the SRFT Mark Holland Metabolic Unit are consented into an ongoing, international, observational database that monitors natural history and outcomes of patients with FD (ClinicalTrials.gov identifier: NCT00196742). The study was conducted in full conformity with the Declaration of Helsinki and with Research Governance Framework and Good Clinical Practice. Consent for the present study was not sought from individual patients as all analyses were of anonymized data from the existing database and from the SRFT PACS. The protocol was approved by the National Research Ethics Service Committee West Midlands–South Birmingham and host institution.

## Results

Of the 149 included participants, only a minority were hypertensive or diabetic, and both mean eGFR and total cholesterol at baseline were within normal limits (table 1). Most participants were on ERT between their baseline and final MRI and had experienced peripheral pain previously. Seventy-six percent of patients with a history of peripheral pain were on ERT while 55% of patients without a history of peripheral pain were on ERT; “peripheral pain at any point” was associated with “ERT at any time during study” on unifactorial analysis (χ^2^ test, *p* = 0.01).

**Table 1:**
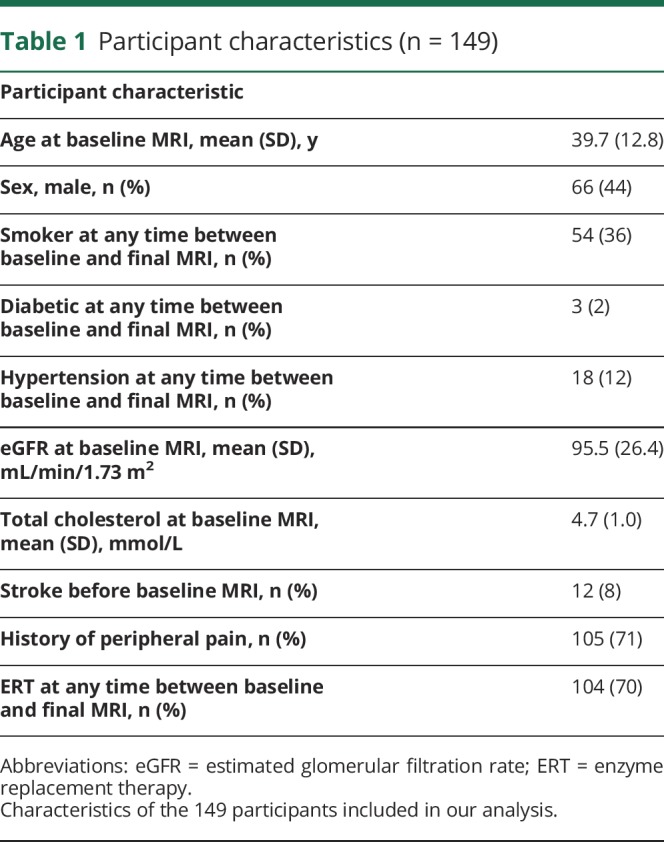
Participant characteristics (n = 149)

**Figure 2:**
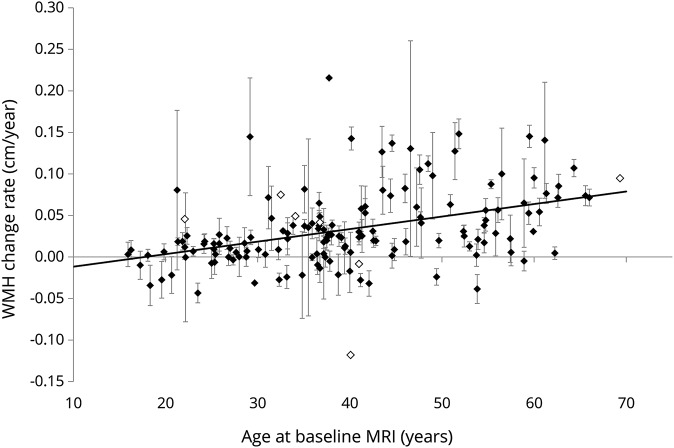
WMH change rate increases with age in Fabry disease Each data point represents an individual patient with Fabry disease (n = 149). WMH change rate is the rate of change (cm/y) produced by linear regression performed on data from each participant in which the dependent variable was the cube root of total WMH volume (cm^3^) at each MRI time point and the independent variable was the time (years) from baseline MRI to each MRI time point. Black symbols = patients with 3 or more measurements. White symbols = patients with 2 measurements. Error bars represent the standard error of the WMH change rate estimate, obtained from the linear regression. It is not possible to calculate the error of 2-point estimates using this method. WMH = white matter hyperintensity.

All patients were found to have WMH; median (IQR) WMH volume at baseline MRI was 1.58 (1.57) cm^3^ in the entire study cohort. Mean (SD) change in cube root of (total WMH volume in cm^3^) per year, which we abbreviate to WMH change rate, was 0.033 (0.047) cm/y in the entire study cohort. Figure 2 shows the WMH change rate for each of the 149 included participants plotted against their age at baseline MRI. A negative WMH change rate was found in 24 patients (16%).

Characteristics associated with WMH change rate on unifactorial analysis (table 2) were age at baseline MRI (*p* < 0.0005; increasing age associated with faster WMH progression), eGFR at baseline MRI (*p* < 0.0005; increasing eGFR associated with slower WMH progression), and treatment with “ERT at any time between baseline and final MRI” (*p* < 0.0005; ERT associated with faster WMH progression). None of the remaining characteristics were significantly associated with the primary outcome after Bonferroni correction for multiple comparisons.

**Table 2:**
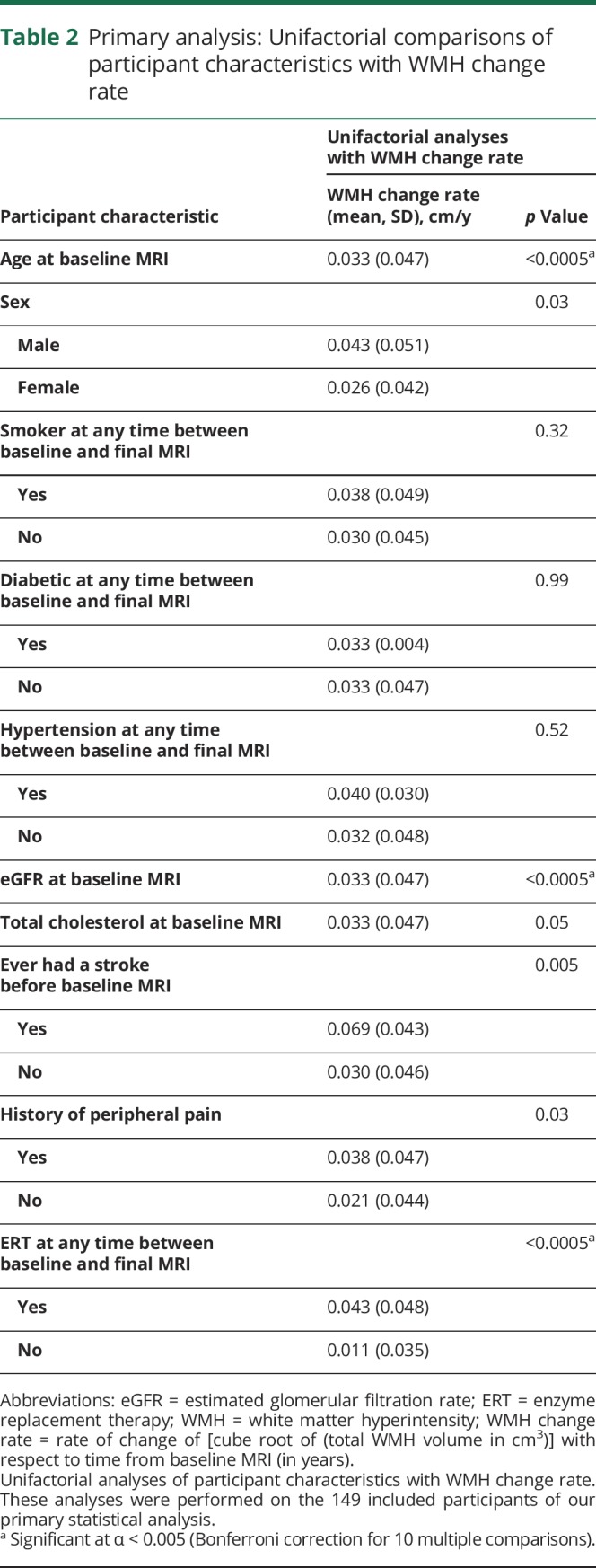
Primary analysis: Unifactorial comparisons of participant characteristics with WMH change rate

On multiple linear regression (table 3), age at baseline MRI (*p* < 0.0005; increasing age associated with faster WMH progression), total cholesterol at baseline MRI (*p* = 0.03; increasing total cholesterol associated with slower WMH progression), and a history of peripheral pain (*p* = 0.02; peripheral pain associated with faster WMH progression) were associated with WMH change rate. None of the remaining characteristics were significantly associated with the primary outcome on multiple linear regression. There were no tolerance values <0.5 and no variance inflation factor values >1.8 (data available from Dryad, table e-3, doi.org/10.5061/dryad.9271vq7), suggesting against significant collinearity between independent variables in the primary statistical analysis.

**Table 3:**
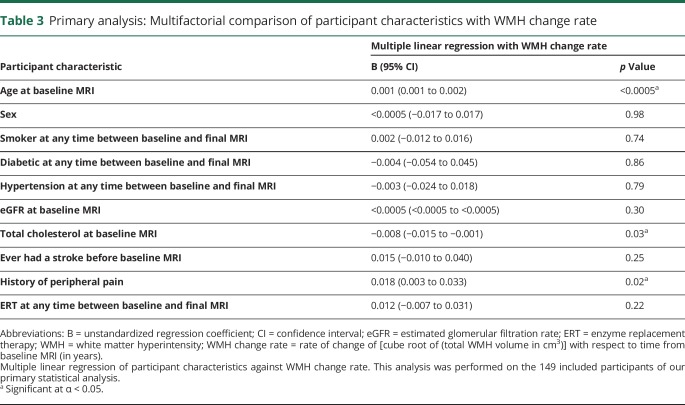
Primary analysis: Multifactorial comparison of participant characteristics with WMH change rate

For all included patients, mean (SD) duration of ERT treatment at final MRI was 63 (56) months. In post hoc analysis 1 (table 4), increasing duration of ERT treatment at final MRI was associated with faster WMH progression (*p* = 0.045). Age at baseline MRI, total cholesterol at baseline MRI, and a history of peripheral pain remained significantly associated with WMH change rate, as per the primary statistical analysis.

**Table 4:**
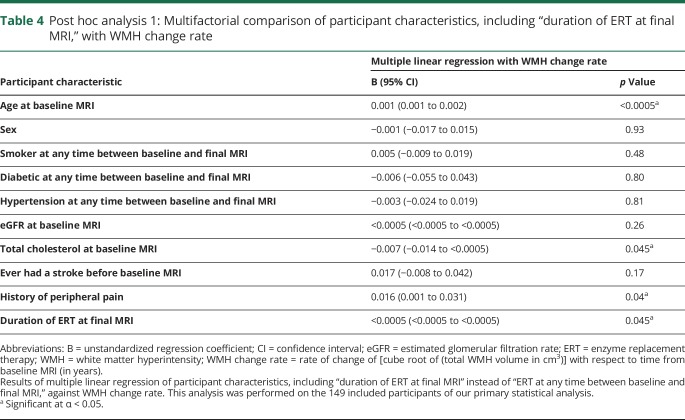
Post hoc analysis 1: Multifactorial comparison of participant characteristics, including “duration of ERT at final MRI,” with WMH change rate

In post hoc analysis 2, 37% (43/117) had received statins between their baseline and final MRI. Of the 43 statin users, 28% (12/43) had a stroke before their baseline MRI while 72% (31/43) had not. All patients who had a stroke before their baseline MRI were receiving a statin. On multiple linear regression (table 5), the following characteristics were found to be associated with WMH change rate: age at baseline MRI (*p* < 0.0005; increasing age associated with faster WMH progression); total cholesterol at baseline MRI (*p* = 0.03; increasing total cholesterol associated with slower WMH progression); history of peripheral pain (*p* = 0.01; peripheral pain associated with faster WMH progression); and “statin–no stroke” (*p* = 0.004; slower WMH progression in participants who had not had a stroke before baseline MRI and were on a statin relative to participants who had not had a stroke and were not on a statin). ERT between baseline and final MRI remained nonsignificant.

**Table 5:**
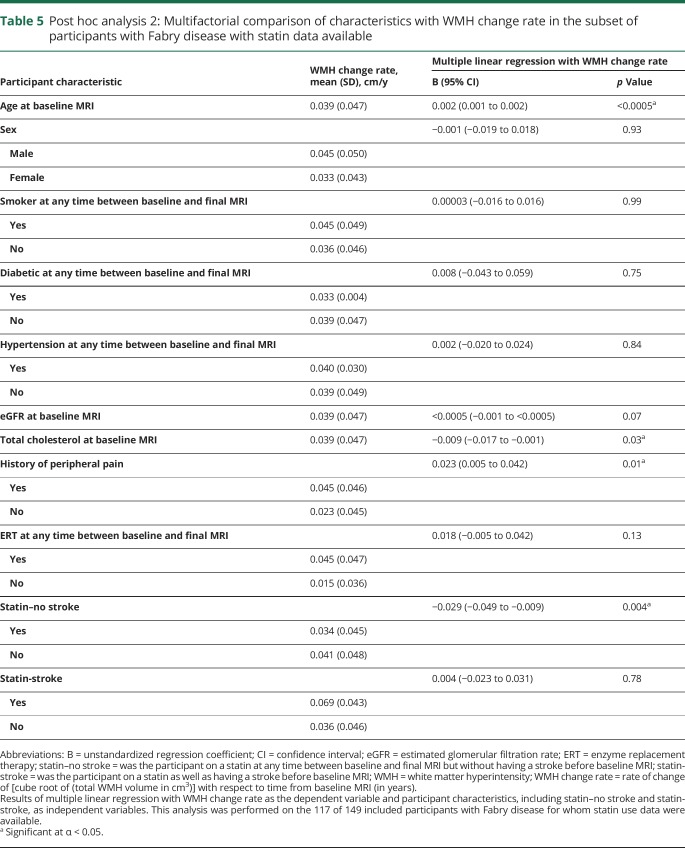
Post hoc analysis 2: Multifactorial comparison of characteristics with WMH change rate in the subset of participants with Fabry disease with statin data available

In post hoc analysis 3, of the 81 (out of 149) included participants who were younger than 40 years at baseline (data available from Dryad, table e-4, doi.org/10.5061/dryad.9271vq7), only total cholesterol at baseline MRI was associated with WMH change rate on multiple regression (*p* = 0.049; increasing total cholesterol associated with slower WMH progression).

In post hoc analysis 4 (data available from Dryad, table e-5, doi.org/10.5061/dryad.9271vq7), the inclusion of “baseline total WMH volume” as an independent variable in multiple regression did not alter the significance or direction of any of the statistical associations between the other participant characteristics and WMH change rate, and “baseline total WMH volume” was not associated with WMH change rate (*p* = 0.52).

In post hoc analysis 5, scan orientation (*p* = 0.25), voxel volume (*p* = 0.71), and interslice gap (*p* = 0.71) were not associated with the cube root of the WMH volume of the baseline MRI scan on multiple linear regression.

## Discussion

We did not find an association between treatment with ERT and rate of WMH progression in patients with FD. This was also the case in a post hoc subgroup analysis of participants younger than 40 years at baseline. While increasing duration of ERT treatment at final MRI was associated with faster WMH progression in post hoc analysis 1, it is possible that this results from patients on ERT for longer having a more severe disease phenotype, rather than because ERT accelerates WMH progression. At the very least, our results do not support a protective role for ERT against WMH progression, even when considering treatment duration. There are multiple reasons why ERT might not influence WMH progression in FD, including the inability of ERT to cross the blood-brain barrier.^19–21^

Our findings are in contrast to a phase IV randomized controlled trial of ERT vs placebo in 41 patients with FD,^8^ which found that ERT stabilized WMH progression over at least 12 months of follow-up in younger participants (<50 years). Furthermore, prior ERT exposure has been associated with reduced WMH volume in 31- to 40-year-old patients on cross-sectional analysis.^10^ Other previous studies evaluating the rate of WMH progression while on ERT have reported conflicting results.^3,5,30–32^ While a randomized controlled trial would be needed to test definitively the relationship between ERT and WMH progression, such a trial might not be achievable given the widespread use of ERT in clinical practice and the lack of clinical equipoise regarding the efficacy of ERT in treating cardiac or renal complications of FD. Our large sample size (n = 149), large number of MRI time points analyzed (n = 863) with at least 2 years of follow-up per participant, and analysis of the rate of WMH progression while patients with FD were on ERT therefore adds substantially to the existing literature.

Our cohort of included participants was relatively young (mean 39.7 years) and had a relatively low incidence of cardiovascular risk factors. Nevertheless, 8% had a previous stroke, which is a similar proportion to previous studies.^10^ Contrary to previous studies,^8,10^ we found no association between history of stroke and WMH progression in FD. Furthermore, we found no association between hypertension, smoking, or diabetes and WMH progression in FD. This suggests that the relationship between “typical” vascular risk factors, stroke, and cerebral small vessel disease in FD is not as well understood as their relationship in the general population.

Increasing age and a history of peripheral pain were associated with faster WMH progression on multivariate analysis. Age has been associated with WMH previously.^6,10^ We have interpreted the association between peripheral pain and WMH progression as the result of peripheral pain representing a more severe FD phenotype that concomitantly causes cerebrovascular pathology and thus WMH progression.

An unexpected finding from the present study is that increasing total cholesterol at baseline MRI was associated with slower WMH progression, even after controlling for statin use in post hoc analysis 2. Of note, participants who were stroke-free and on a statin also had a slower rate of WMH progression than stroke-free participants not on a statin. The apparent protective effect of total cholesterol on WMH progression might theoretically be due to “survivor bias,” namely, that “typical” patients with hypercholesterolemia might have more cardiovascular disease and therefore be lost to follow-up because of comorbidity or death, leaving “atypical” patients with hypercholesterolemia who have other factors protecting against vascular disease. However, within the limitations of the present study (including that nonfasting lipid samples were used), these results raise the possibility that there might be a direct protective effect of high total cholesterol on cerebral small vessel disease in FD. These results also suggest that statins might protect against WMH progression in patients with FD who have not previously had a stroke, and that this protective effect might not be mediated by influencing total cholesterol levels. While this relationship has not been found previously in FD^33^ or in young stroke patients without FD,^34^ a similarly counterintuitive protective effect of cholesterol on risk of hemorrhagic stroke has been described.^35^ The present study suggests that the complex relationship between cholesterol, statin use, and cerebral small vessel disease in FD should be investigated further.

Of interest, 16% of included participants had a negative WMH change rate, implying that WMH volume might decrease over time in a minority of patients with FD. While WMH have been historically viewed as representing irreversible ischemic damage, it has been suggested from case reports of hepatic encephalopathy^36^ and carotid artery stenting^37^ that they may also represent reversible cerebral edema. Indeed, WMH regression has been observed to occur in a significant proportion of patients with ischemic stroke over intervals as short as 6 months^38^ to 1 year.^39^ Considering the error bars on figure 2, it can be seen that for approximately half of the participants with a negative WMH change rate, the finding is robust, showing a significant reduction in WMH volume over time.

This study has several limitations. First, there was variability in the MRI scanners and image acquisition protocols used over the 10-year period from which data were collected. However, post hoc analysis 5 failed to find an association between 3 key scanning parameters and the cube root of baseline WMH volume, suggesting against scanning parameter variability acting as a significant confound in the primary statistical analysis. Furthermore, as MRI scanning parameters should be distributed randomly among study participants, such random variation in MRI scanners and image acquisition parameters used should only increase random error in the WMH change rate, rather than introduce systematic bias.

A second limitation is that, because of a lack of a high-resolution T1-weighted image corresponding to each T2-FLAIR image, we did not coregister MRIs to baseline scans. The resultant partial volume effects are likely to be a source of random variation in the calculated WMH volumes and add to the measurement error. However, as with random variation in the image acquisition parameters, we would expect that such partial volume effects would be distributed randomly throughout different time points and therefore should increase random error in the WMH change rate, rather than introduce systematic bias.

In this observational study, the nonrandom selection of patients for ERT would be anticipated to confound any relationship between disease status and WMH change rate. Regression diagnostics suggested against significant collinearity between independent variables in the primary statistical analysis (data available from Dryad, table e-3, doi.org/10.5061/dryad.9271vq7), and therefore that it is “peripheral pain at any point” that “wins out” over “ERT treatment at any time during study” in explaining the variation in our outcome variable (WMH change rate). However, it is possible that residual confounding factors exist to influence the observed relationships between the included independent variables and the rate of WMH progression. In particular, there might be systematic differences in disease severity between patients on ERT and those not on ERT that is not fully captured by the measured participant characteristics.

This study did not include a control group. However, healthy controls would not be matched to the FD cohort for ERT status, prevalence of peripheral pain, or (in age-matched controls) prevalence of previous stroke. Furthermore, including a control group would not have changed our failure to find an association between ERT and WMH progression in FD.

We have used WMH progression as a marker of cerebrovascular pathology, but since we did not measure occurrence of new strokes, it is possible that ERT could be associated with stroke incidence independently of WMH progression.

Finally, while we did not find evidence for an association between ERT and WMH progression, this is logically distinct from direct evidence that they are not associated; our results should be interpreted accordingly.

## Publication history

Received by *Neurology* September 21, 2017. Accepted in final form July 12, 2018.

## Glossary

eGFR: estimated glomerular filtration rate
ERT: enzyme replacement therapy
FD: Fabry disease
FLAIR: fluid-attenuated inversion recovery
IQR: interquartile range
PACS: Picture Archive Communication System
SRFT: Salford Royal Foundation Trust
WMH: white matter hyperintensity
